# Endobronchial Hamartoma Misdiagnosed as Pneumonia: A Case Report

**DOI:** 10.1002/ccr3.71528

**Published:** 2025-11-27

**Authors:** Lina Peng, Cuixia Bian, Ran Gao, Baowei Sheng

**Affiliations:** ^1^ Department of Respiratory and Critical Care Medicine Jining First People's Hospital Jining Shandong People's Republic of China; ^2^ Departments of Respiratory Medicine Jiaxiang People's Hospital Jining Shandong People's Republic of China

**Keywords:** benign endobronchial tumor, bronchoscopic resection, case report, endobronchial hamartoma, pulmonary hamartomas

## Abstract

Endobronchial hamartomas are rare benign bronchial tumors. We describe the case of a 43‐year‐old man with a cough and fever for 2 months. Initially, the patient was misdiagnosed with obstructive pneumonia caused by a bronchial sputum thrombus, and repeated treatments with antibiotics and mechanical expectoration were unsuccessful. Bronchoscopy revealed that the mass had completely obstructed the lower lobe of the left lung. The electrocautery snare technique was used to completely resect the lesion. Histopathological examination confirmed the diagnosis of hamartoma. This case serves as an important reference for clinical practice to prevent future misdiagnoses.

**Key Clinical Message**

Endobronchial hamartoma is a rare, benign tumor that is often misdiagnosed or overlooked in clinical practice. If a patient's pneumonia does not improve after treatment, it is important to promptly perform bronchoscopy to rule out airway obstruction caused by the hamartoma in the bronchi.

## 1 Introduction

Hamartomas are the most prevalent benign tumors, with a total incidence rate of 0.025% to 0.32% [1]. Most pulmonary hamartomas involve the lung parenchyma. Approximately 10% of pulmonary hamartomas are bronchial hamartomas, which are notably uncommon [2]. The clinical signs and symptoms of pulmonary parenchymal hamartomas are often subtle. Endobronchial hamartomas can produce respiratory symptoms such as coughing, breathing problems, fever, and even haemoptysis because they restrict the airways. Endobronchial hamartomas can be diagnosed using computed tomography (CT); however, misdiagnosis is common in clinical practice because of the lack of distinct CT images or specific clinical symptoms associated with bronchial hamartomas. Bronchoscopy is considered the gold standard for diagnosing endobronchial hamartomas. Recently, various treatment options have emerged for endobronchial hamartomas. In addition to conventional surgical lobectomy, bronchoscopy employs various methods, such as cryotherapy, argon plasma coagulation, and electrosurgical snare techniques, which are not only highly effective but also safe [3]. This article describes a middle‐aged man who was initially misdiagnosed with obstructive pneumonia due to sputum thrombosis. Despite undergoing repeated treatments over the course of 2 months, the patient showed no improvement. It was not until he received a bronchoscopic electrosurgical snare that the underlying pathology became clear, and his health began to improve.

## 2 Case History/Examination

The patient is a 43‐year‐old man with no history of chronic illnesses. He had no history of smoking, alcohol consumption, or exposure to chemicals. He was admitted to the respiratory clinic of our hospital because of dry cough and fever lasting for 2 months. Over this period, he experienced recurrent episodes of dry cough and fever, with his body temperature reaching as high as 38.5°C. The patient did not report any additional symptoms such as diarrhea, frequent urination, chest pain, or common cold. No pathogenic bacteria were detected in the sputum cultures. A CT scan performed at a local hospital revealed obstructive pneumonia and a sputum thrombus in the bronchus of the lower lobe of the left lung. Pneumonia was considered during a pulmonary consultation at a local hospital. The consultation recommended anti‐infection and phlegm treatments and an elective review of chest CT.

## 3 Methods (Differential Diagnosis, Investigations, and Treatment)

**FIGURE 1:**
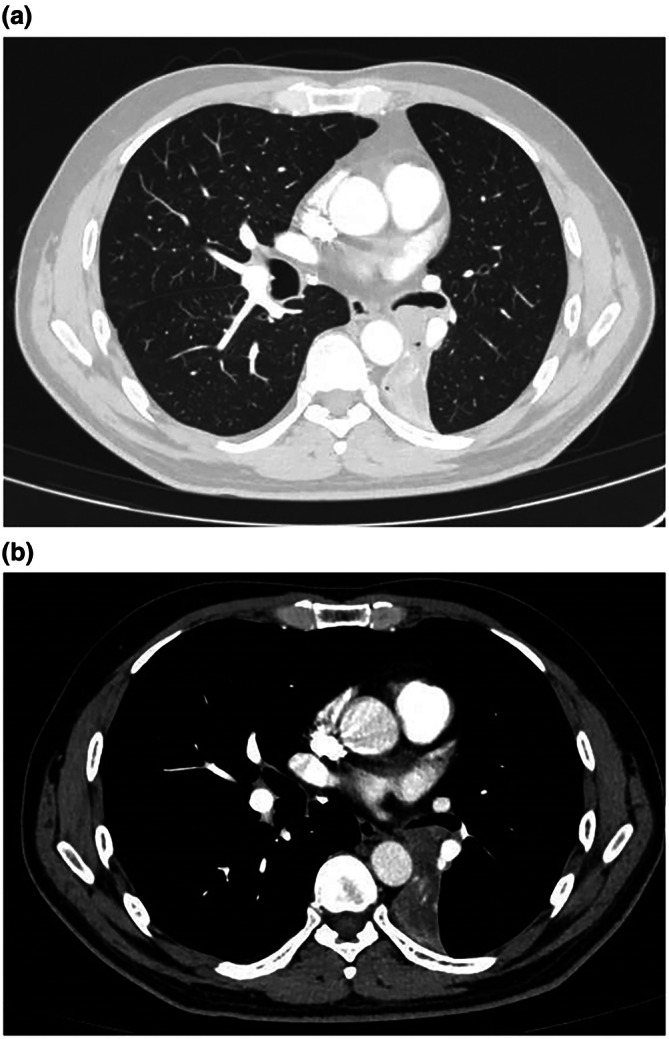
Chest‐enhanced CT scan revealing a 1.9 × 1.3 cm nodular lesion causing consolidation and atelectasis of the left lower lobe. (a) Lung window. (b) Mediastinal window.

Based on the patient's clinical symptoms and the results of the chest CT scan, the local hospital established a preliminary diagnosis of pneumonia and sputum thrombus in the bronchus. For 2 weeks, the patients received mechanically assisted expectoration, antibacterial, and antitussive therapy. Although the patient's fever and cough subsided, they recurred. Following admission to our hospital's respiratory department, a full chest enhanced CT scan revealed a 1.9 × 1.3 cm nodular lesion near the origin of the bronchus in the lower lobe of the left lung, with no significant enhancement, along with consolidation and atelectasis in the same region (Figure 1). Lung cancer indicators such as CEA, CF21‐1, NSE, and SCC were all within the normal range. The patients' symptoms and imaging findings were considered when assessing the risk of bronchial masses and foreign bodies. Therefore, bronchoscopy was performed.

**FIGURE 2:**
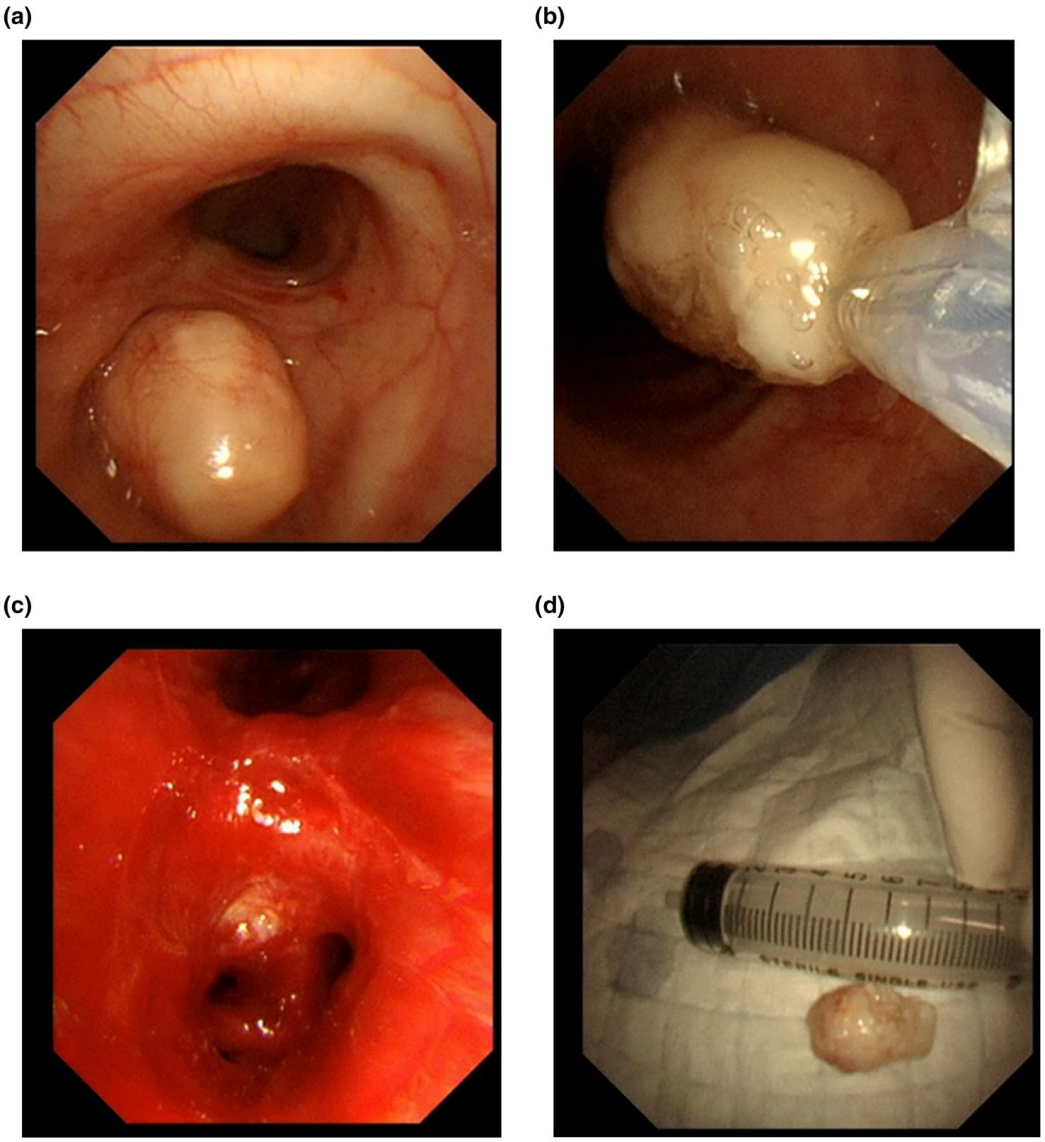
Bronchoscopic electrocautery snare treatment. (a) A smooth mass completely blocking the lower lobe of the left lung. (b) Electrocautery snare treatment. (c) The unobstructed lower lobe of the left lung after treatment. (d) The resected mass.

The bronchoscopy procedure was conducted under general anesthesia. To ensure an adequate supply of oxygen, we chose to use high‐frequency ventilation for the patient. Bronchoscopy revealed a mass that completely blocked the entrance to the lower lobe of the left lung. The mass had a broad base, a smooth surface, and a stalk firmly attached to the bronchial opening of the lower lobe. We switched to a rigid bronchoscopy to prevent the removal of the lump from blocking the glottis. After several treatments using an electrocautery snare under rigid bronchoscopy, the mass (approximately 2 cm in length) was successfully resected. There was a small amount of bleeding during the resection of the mass, which we successfully managed by locally spraying epinephrine. There was significant purulent discharge from the lower lobe of the left lung. After multiple rounds of aspiration and irrigation, the lower lumen of the left lung was cleared (Figure 2).

## 4 Conclusion and Results (Outcome and Follow‐Up)

**FIGURE 3:**
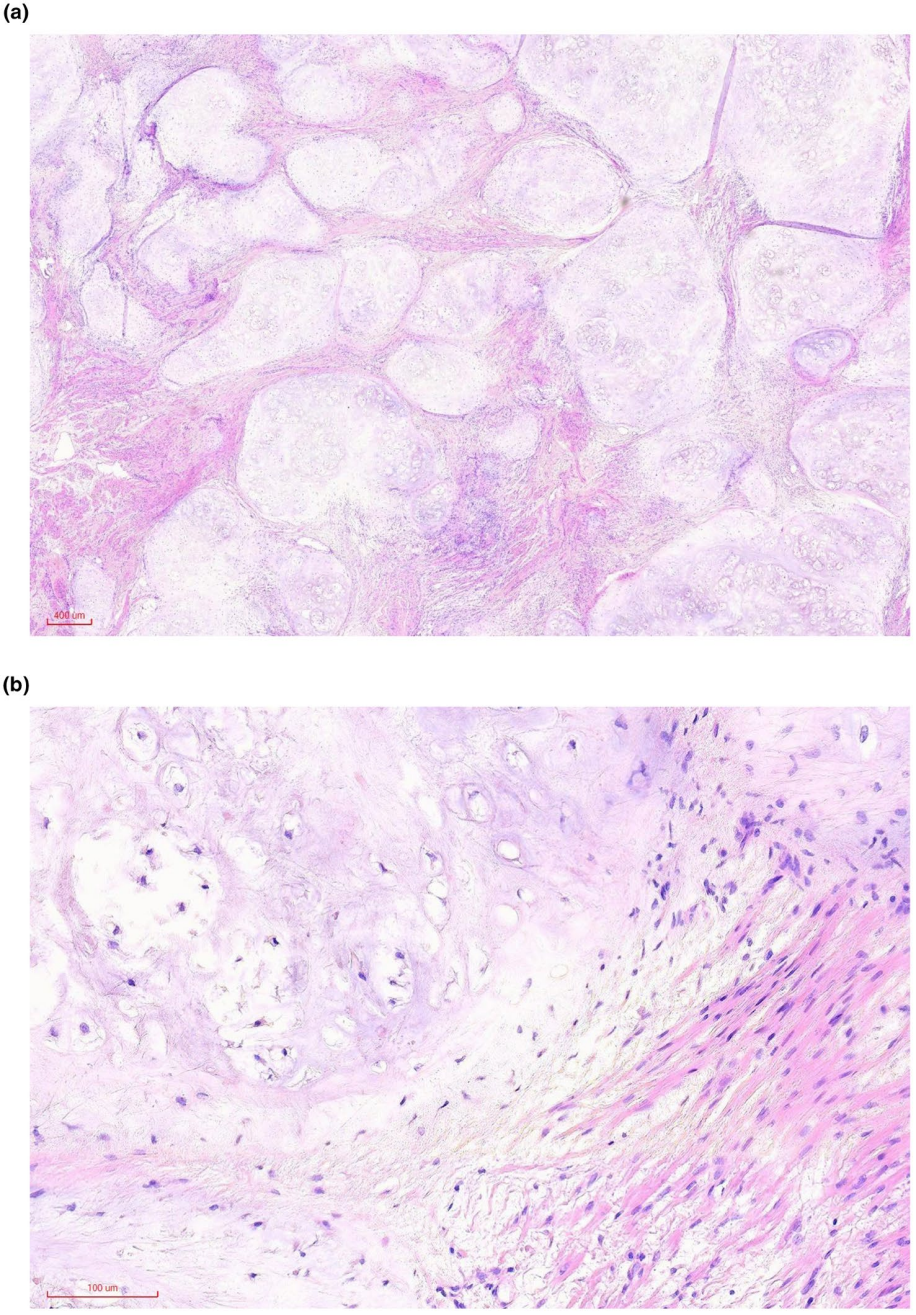
Histopathology of the tumor resected via bronchoscopy. Hematoxylin‐eosin staining. (a) Low‐magnification view. (b) High‐magnification view.

**FIGURE 4:**
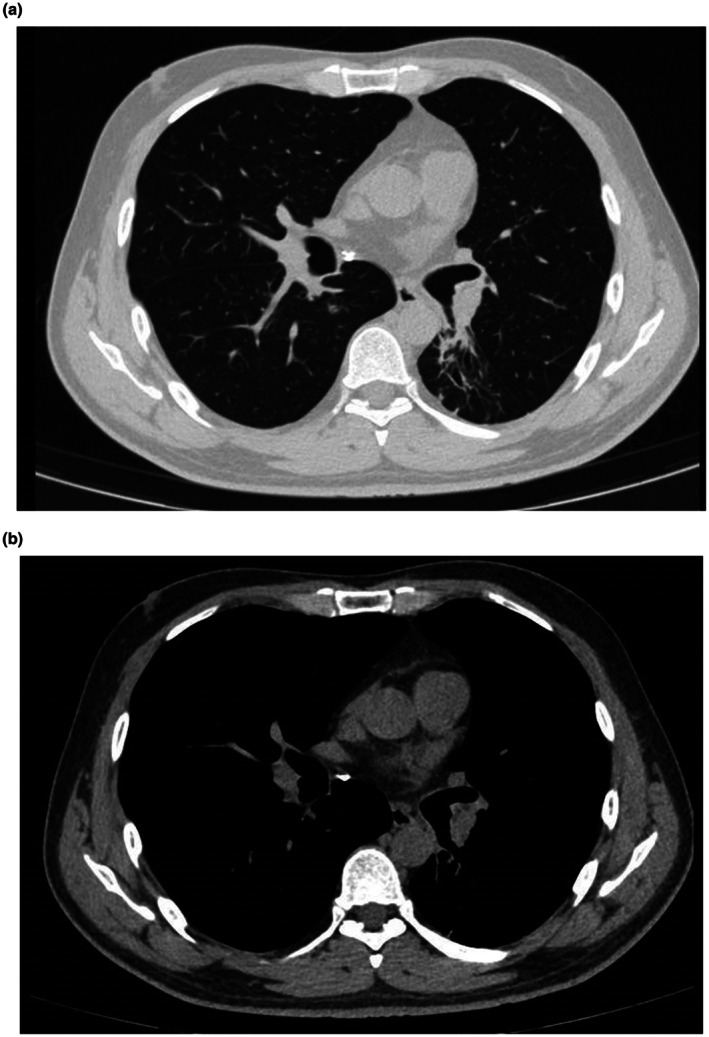
Follow‐up chest CT showing the airways unobstructed and the left lower lung re‐expanded. (a) Lung window. (b) Mediastinal window.

Following bronchoscopy, the patient's cough significantly improved, and the fever subsided. Pathological examination confirmed that the excised mass was a hamartoma (Figure 3). A follow‐up chest CT performed a week after the procedure showed that the left lower lung had re‐expanded, and the airways in the lower lobe were unobstructed (Figure 4). The patient was discharged from the hospital smoothly and is currently undergoing routine chest CT follow‐up at our hospital's outpatient department. The hamartoma did not recur.

## 5 Discussion

Benign endobronchial tumors are uncommon in clinical practice. Approximately 39% of benign endobronchial tumors are hamartomas (11/28) [4]. Histologically, hamartomas frequently contain several components. Endobronchial hamartomas typically contain fibroblasts, fat, and cartilage [5]. The detection rate of rare endobronchial hamartomas has recently increased owing to ongoing advancements in bronchoscopy, and physicians must pay close attention to their diagnosis and treatment.

Pneumonia symptoms, such as fever, cough, and dyspnoea, are often caused by endobronchial hamartomas. These lesions typically present as space‐occupying masses in the soft tissues of the bronchi on imaging studies and can lead to atelectasis [6]. On CT, these lesions usually appear spherical or nearly circular, have calcifications resembling popcorn, and are fat‐dense [7]. In the present case, the patient experienced chronic cough and fever. The CT scan of the lesion in the left lower lung bronchus did not show typical signs of hamartomas, such as fat density or popcorn‐like calcification. Instead, the scan indicated that the sputum had obstructed the bronchus of the left lower lung. Initially, the condition was misdiagnosed as pneumonia or bronchial sputum thrombus. After more than 2 months of unsatisfactory treatment, a thorough bronchoscopy confirmed the diagnosis, which highlights two important insights. First, when encountering similar cases in clinical practice, rare disorders, such as bronchial hamartoma, should be considered to avoid missed or incorrect diagnoses. Second, despite the associated risks, bronchoscopy should be performed proactively, alongside discussions with patients.

Both surgical and endoscopic procedures are used to treat endobronchial hamartomas. Abdel Hady [8] et al. compared the advantages and disadvantages of surgical and bronchoscopic resection for these tumors. Their findings showed that bronchoscopic resection had favorable outcomes with fewer complications and eliminated the need for lung resection. Therefore, endoscopic treatment is the preferred approach for endobronchial hamartomas. According to Song et al., treatment outcomes were favorable and there were no treatment‐related complications, such as major haemoptysis, airway perforation, or hypoxia, among 13 patients with endobronchial hamartomas who were treated using bronchoscopic intervention [3]. Several methods, including cryosurgery [9], laser and microwave ablation [10], and electrocautery snare [11] under bronchoscopy, can be used to excise airway tumors. In conclusion, the best endoscopic treatment option can be selected based on the location, size, and shape of the lesion. In this case, microscopic findings revealed a smooth, pedunculated tumor that completely obstructed the lower lobe of the left lung. The use of an endoscopic electrocautery snare was appropriate in this situation. There were no visible bleeding issues, and the lesion was completely resected after several treatments using an endoscopic electrocautery snare. Follow‐up CT showed that the airway was unobstructed, the atelectasis had significantly improved, and the patient's symptoms had markedly improved. Moreover, there was no tumor recurrence. These results support those of previous studies, confirming that bronchoscopy is a safe and effective treatment option for managing endobronchial hamartoma with a favorable prognosis.

## 6 Conclusion

The clinical incidence of endobronchial hamartoma is low, and its clinical symptoms and imaging findings are often nonspecific. This can lead to misdiagnoses or missed diagnoses in clinical practice. However, early diagnosis and prompt treatment can significantly enhance prognosis. This case serves as an important reference for the diagnosis and management of endobronchial hamartomas.

## Author Contributions

**Lina Peng:** investigation, writing – original draft. **Cuixia Bian:** supervision, writing – review and editing. **Ran Gao:** resources, writing – original draft. **Baowei Sheng:** data curation, writing – review and editing.

## Funding

The authors have nothing to report.

## Consent

Written informed consent was obtained from the patient.

## Conflicts of Interest

The authors declare no conflicts of interest.

## Data Availability

Data sharing is not applicable to this article, as no datasets were generated or analysed in the current study.
